# A non-radioactive method for small RNA detection by northern blotting

**DOI:** 10.1186/s12284-014-0026-1

**Published:** 2014-10-01

**Authors:** Qi Huang, Zhinang Mao, Shaoqing Li, Jun Hu, Yingguo Zhu

**Affiliations:** State Key Laboratory of Hybrid Rice, College of Life Sciences, Wuhan University, Wuhan, 430072 China; Engineering Research Center for Plant Biotechnology and Germplasm, Utilization, Ministry of Education, Wuhan University, Wuhan, 430072 China; Suzhou institute of Wuhan University, Wuhan, 215000 China

**Keywords:** Small RNA, Biotin-labeled probe, Non-radioactive, Northern blot, Epigenetic

## Abstract

**Background:**

Small non-coding RNAs are essential regulators of gene expression at the transcriptional and posttranscriptional levels. High-throughput sequencing has revealed thousands of predicted small RNAs; however, only a few of these have been well characterized. Northern blotting is the most convincing method for small RNA validation.

**Findings:**

In this study, we improved the Northern blot method by using biotin-labeled probes. miRNAs and siRNAs derived from both *Arabidopsis thaliana* and *Oryza sativa* were investigated. The results suggest that this improved method is sensitive and efficient, with approximately 5 μg of total RNA being sufficient for detection. Furthermore, long-term storage of probes labeled in this manner is more convenient, less contaminative and degradative compared with traditional probes.

**Conclusions:**

This protocol is an alternative strategy for small RNA detection and represents an efficient means of researching small RNAs.

**Electronic supplementary material:**

The online version of this article (doi:10.1186/s12284-014-0026-1) contains supplementary material, which is available to authorized users.

## Findings

In eukaryotic cells, small non-coding RNAs have been demonstrated to play fundamental roles in gene expression modification during development (Zamore & Haley, [2005]). Small non-coding RNAs are 20-30 nt long and function as sequence-specific negative regulators of gene expression at the transcriptional and/or posttranscriptional levels (He & Hannon, [2004]; Voinnet, [2009]). The three major types of small non-coding RNAs—miRNAs, siRNAs, and piRNAs—are distinguished by their different modes of biogenesis (Chen, [2009]; Huang et al, [2011]). In plants, miRNAs and siRNAs are the two major classes of endogenous small RNAs and are important for development, genome stability, gene expression and stress responses (Chen, [2012]; Wei et al, [2012]). Although the mechanisms and pathways by which miRNAs and siRNAs regulate their target genes are largely obscure, the initial findings have demonstrated that miRNAs and siRNAs have a broad impact on epigenetics.

Since the discovery of miRNAs in *C. elegans* two decades ago, remarkable advances in the characterization of this gene family have been achieved (He & Hannon, [2004]). Recently, high-throughput sequencing technology has facilitated the exploration of small non-coding RNAs (Creighton et al, [2009]; Studholme, [2012]). To date, thousands of small non-coding RNAs and their target mRNAs or genes have been computationally predicted; however, few of them have been experimentally confirmed or properly characterized. To gain further insight, several methods have been developed to investigate the expression of target small non-coding RNAs, such as Northern blotting, quantitative reverse-transcription PCR (RT-PCR), and in situ hybridization.

rRNAs and tRNAs account for over 90% of the total RNA in a eukaryotic cell, whereas small non-coding RNAs account for only approximately 2%, and detection of these RNAs by some methods is difficult (Zhuang et al, [2012]). Because the low abundance of small RNAs can be problematic for detection via northern blotting, some researchers have enriched small RNAs using LiCl or other methods (Song et al, [2012]). Although isotope labeling is often inconvenient, hazardous and restricted by many institutions, this classic method is still the most popular method for investigating the expression of small RNAs. Several improvements have been made to traditional Northern blotting protocols, and non-isotopic-labeling methods using digoxigenin (DIG) represent a safe alternative for the detection of small RNAs (Ramkissoon et al, [2006]). In recent years, researchers have been continuously improving the method for northern blotting with digoxigenin (DIG)-labeled modified probes (Kim et al, [2010]). Researchers have also modified probes with locked nucleic acid (LNA) to improve sensitivity (Gao & Peng, [2011]; Lopez-Gomollon, [2011]).

Biotin is another molecule that can increase the sensitivity of small RNA detection. In this study, we designed biotin-labeled oligonucleotide probes to investigate the expression of small RNAs. When we applied this improved method to *Arabidopsis thaliana* and *Oryza sativa* for validation, the results suggested that this method was sensitive and efficient, as it was capable of detecting as little as 1 to 5 μg of total RNA. Furthermore, storage of biotin-labeled probes is convenient, and the probes are stable; therefore, northern blots can be carried out with ease. Taken together, these data suggest that biotin-labeled probes can be widely used to investigate small RNAs and even mRNAs in the future.

### Preparation of total RNA and small RNA for Northern blotting

**Figure 1:**
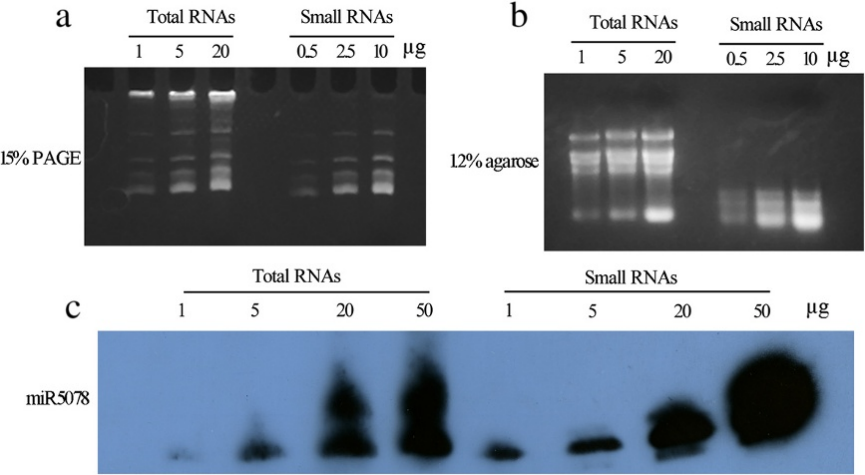
**Integrity of extracted RNAs. (a)** Total RNA (left) and small RNAs (right) were loaded on 15% denaturing PAGE gels and stained with 0.5 μg/ml EtBr in buffer. **(b)** Total RNA (left) and small RNAs (right) were loaded on 1.2% agarose gels and stained with 0.5 μg/ml EtBr in buffer. **(c)** Northern blot of miRNA5078 with variable amounts of total RNA (left) and small RNAs (right). The exposure times are indicated.

In a eukaryotic cell, the abundance of rRNA and tRNA impairs the detection of small RNAs. To test the method of RNA preparation in this study, total RNA and small RNAs were extracted with two different reagents. To detect the integrity of the RNA, we loaded the RNA onto 15% PAGE gels under denaturing conditions (7 M urea) or onto 1.2% agarose gels with EtBr for staining. The results showed that the RNAs were suitable for further analysis, such as northern blotting (Figure 1a,b). Next, the amounts of RNA were quantified using a NanoDrop (Thermoscientific). The data suggested that the small RNAs, including 5S rRNA, tRNA and small non-coding RNAs, accounted for approximately 25% of the total RNA, and three-quarters of the unwanted RNA was removed effectively (Table 1). Hence, this RNA isolation method is suitable for extracting small fragment RNAs and for removing the abundant RNAs. Northern blots were further performed to detect the expression of miRNAs. miRNA5078 was specifically investigated using a miRNA5078-biotin-labeled probe. The data established that miRNA5078 is present in leaves (Figure 1c). This result also indicated that 5 μg of total RNA or 1 μg of small RNA was sufficient for detection.

### Northern blots of miRNA and siRNA of Arabidopsis

**Figure 2:**
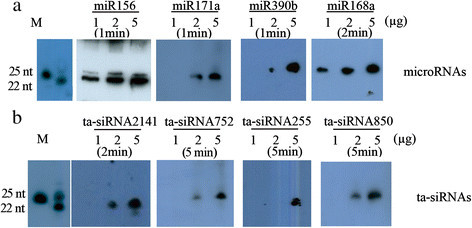
**Detection of miRNAs and ta-siRNAs from** ***Arabidopsis*** **. (a)** Northern blots for miRNAs using biotin-labeled probes and variable amounts of total RNA from *Arabidopsis* leaves. The exposure time is indicated. **(b)** Northern blots for ta-siRNAs using biotin-labeled probes and variant total RNA from *Arabidopsis* leaves. The exposure time is indicated.

Because northern blots performed using biotin-labeled probes were sensitive and efficient, we used this method to confirm the expression of classic miRNAs and siRNAs in *Arabidopsis*. It is well known that the abundance of small non-coding RNAs is less than 2%. To address this problem, researchers usually increase the amount of input RNA to 20–60 μg depending on the expression level of the target miRNA. In this study, the input RNAs (ranging from 1 to 5 μg) were loaded onto 15% denatured PAGE gels before northern blotting to test the sensitivity of this method. We examined certain miRNAs (miR156, miR171a, miR390b, and miR168a) because they had previously been shown to be expressed in leaves (Yang et al, [2006]; Zhan et al, [2012]). As shown in Figure 2a, these miRNAs were easily detected after a 1 to 5 minute exposure time, which was determined based on the typical amount of miRNA *in vivo*. Four gene families have been identified in *Arabidopsis* that produce a number of trans-acting siRNAs (ta-siRNAs) (Allen et al, [2005]). ta-siRNAs, such as tasiR-ARF, also play essential roles in plant development (Williams et al, [2005]). Subsequently, we also evaluated the accumulation of ta-siRNAs, such as ta-siRNA2141, ta-siRNA752, ta-siRNA255, and ta-siRNA850. The results demonstrate that this method is also efficient for studying the expression of ta-siRNAs (Figure 2b). Therefore, we confirmed that both miRNAs ta-siRNAs can be detected by this method with small quantities of total RNA.

### Northern blotting of miRNAs and ta-siRNAs from rice

**Figure 3:**
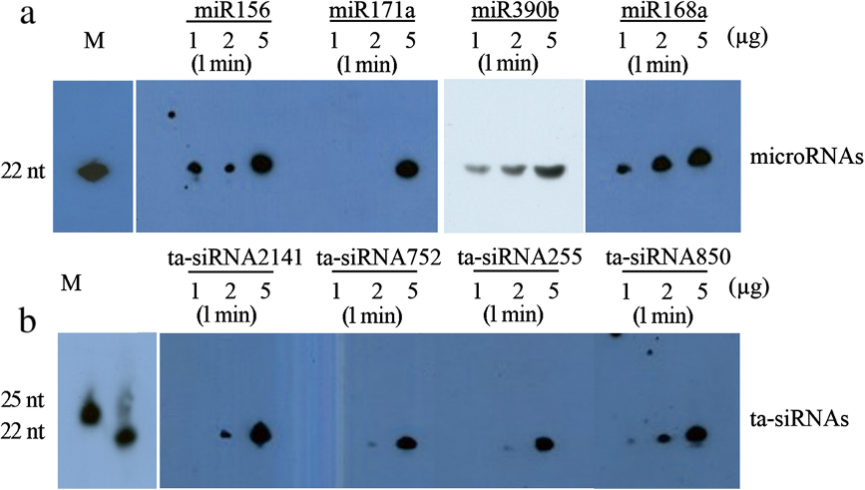
**Detection of miRNAs and ta-siRNAs from rice. (a)** Northern blots for miRNAs using biotin-labeled probes and variant total RNA from rice leaves. The exposure time is indicated. **(b)** Northern blots for ta-siRNAs using biotin-labeled probes and variant total RNA from rice leaves. The exposure time is indicated.

Rice is an important staple crop worldwide, and it is also an essential model crop for research. To ensure that this method can be applied to rice, we also validated established rice miRNAs and ta-siRNAs. Based on previous studies and the Plant MicroRNA Database (http://bioinformatics.cau.edu.cn/PMRD/), miR156, miR171a, miR390b, and miR168a were selected for investigation. As shown in Figure 3a, most miRNAs were easily detected in 5 μg of total RNA with 1 minute of exposure time. Furthermore, the results suggested that detection of ta-siRNA was also successful (Figure 3b). These results demonstrate that this method is an efficient means of assessing the presence of small RNAs in small amounts of total RNA. Indeed, 1 μg of total RNA was sufficient for the detection of some highly expressed small RNAs. For small RNAs with lower expression levels, 5 μg of total RNA was sometimes necessary for detection. Consequently, 1 μg of purified small RNA is sufficient for northern blot applications with this strategy, and this amount can easily be obtained by researchers without any special or expensive equipment.

### Protocol for northern blotting with biotin-labeled probes

**Figure 4:**
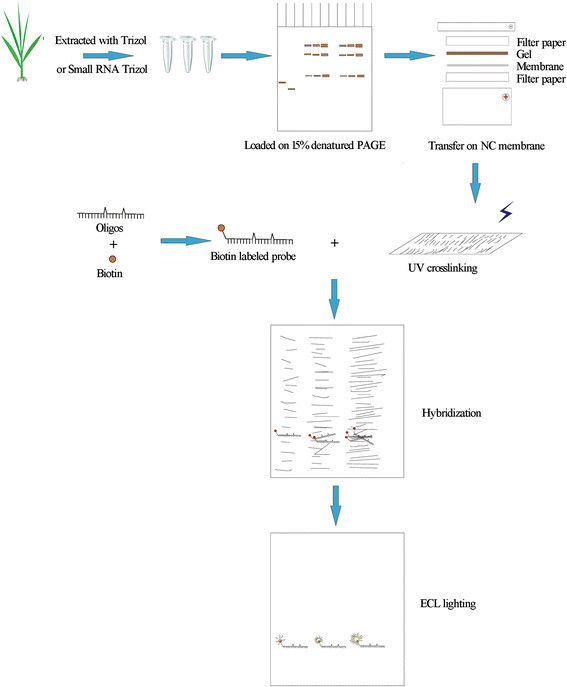
**Schematic diagram of Northern blotting with biotin-labeled probes.**

The methodology described in this study is illustrated in Figure 4. First, the integrity of the RNAs is determined, and the total amount is quantified. Both total RNA and small fragment RNAs can be used for detection, and the amount of the latter needed for detection was only approximately one-quarter of the total RNA. If the precursors of miRNAs or ta-siRNAs were not needed for research, small fragment RNAs were preferred as the loading sample. To avoid degradation of the RNA, the glass plates used for RNA gels should first be wiped with ethanol. It is recommended that the 15% denaturing PAGE gel containing 7 M urea and TBE running buffer be prepared with DEPC treated water. The biotin can be modified on either the 5′ or 3′ terminus, as both methods are available. Probes can be stored as 100 μM stocks at 4°C for 6 months or at -20°C for long-term storage. The complete protocol requires only two days, including overnight hybridization on the first day.

## Discussion

The massive increase in the number of small RNAs being uncovered in numerous organisms increases the need for improved methods of small RNA detection. The overwhelming majority of studies on small RNAs suggest that northern blotting is the most popular method for studying the expression of target small RNAs. However, isotope contamination is a serious problem for the environment and researcher safety, and most researchers are eager to find a safer alternative. Digoxigenin, which is widely used for Southern blots, northern blots, and in situ hybridization, may represent a safe alternative label. We also acknowledge the discovery of biotin, which makes such research easier and more convenient.

In addition to isotope safety, sensitivity is another problem associated with northern blotting. Several improved technologies have been reported, including the use of chemical cross-linking instead of UV, the use of an LNA primer as a probe (Valoczi et al, [2004]), LED (LNA, EDC, DIG) modification (Kim et al, [2010]) and purification and loading of only the small RNA fraction (Song et al, [2012]). Improving the sensitivity of these methods is important, as the amount of miRNA or siRNA is often very low. Biotin is becoming more widely used in biotechnological applications. The affinity of biotin for streptavidin is one of the strongest and most stable non-covalent interactions. Horseradish Peroxidase (HRP) is an enzyme that utilizes organic peroxide compounds as electron donors. The biotin-streptavidin-HRP conjugation method is ideal for investigators working in the fields of immunology, cancer, and neuroscience as well as other areas.

In this study, we significantly improved the northern blotting procedure for detecting small RNAs with the use of a biotin-labeled probe. Biotin-labeled probes have the benefits of being safer and easier to handle and store compared with traditional probes. A biotin-labeled probe can be stored for at least 6 months at 4°C, whereas a ^32^P-labeled probe can be stored for no more than 1 month. Furthermore, biotin-labeled probes are synthesized once and can be re-used more than 100 times, whereas isotope-labeled probes must be re-labeled with every use. Probe sensitivity is extremely important, and the sensitivity of biotin-labeled probes is acceptable based on our studies. Typically, 20-40 μg total RNA is used for northern blots, and even for rice, some researchers still use 30 μg of small fragment RNAs or total RNA for detection by isotope-labeled probes (Wang et al, [2004]; Zhong et al, [2013]). When using biotin-labeled probes, the amount of input RNA can be decreased to 2-5 μg based on our research. If the amount of a particular target small RNA is very low, we recommend increasing the amount of total RNA to 20 μg and the amount of small fragment RNAs to 5 μg. Biotin probes can also save time, as only two days are needed to complete the procedure, and there is no need to spend time labeling or purifying isotopes. The results from this study using both *Arabidopsis* and rice suggest that this method works well and may be suitable for use in other species.

## Conclusions

To date, high-throughput sequencing has revealed thousands of predicted small RNAs; however, only a few of them have been characterized. Most researchers use isotope-labeled probes for small RNA detection and validation. Although biotin has been used in many fields, Northern blots using biotin-labeled probes have not been reported. In this study, we improved Northern blotting with the use of biotin-labeled probes. Northern blots using RNA derived from both *Arabidopsis* and *Oryza sativa* were performed, and the results suggest that this improved method is both sensitive and efficient. Furthermore, biotin-labeled probes are more stable and safer for researchers and the environment than traditional probes. This protocol represents an important contribution to the small RNA field.

## Materials and methods

### Probe Preparation

Some of the miRNAs and ta-siRNAs selected for validation are listed in the small RNA database (http://bioinformatics.cau.edu.cn/PMRD/), and some have been reported in previous studies (Allen et al, [2005]; Xie et al, [2006]; Zhan et al, [2012]). Probes modified with biotin on the 5′ or 3′ terminus were synthesized and purified via HPLC by the GenScript Company (Nanjing, China). These probes were designed to be completely complementary to the miRNA or ta-siRNA nucleotide sequence and are listed in Additional file 1: Table S1.

### RNA extraction

RNA was extracted from the leaves of *Arabidopsis* and *Oryza sativa*. Total RNA was extracted from leaves using TRIzol reagent (Invitrogen, USA) following a previously described protocol (Hu et al, [2012]). Small RNAs were extracted from leaves using RNAiso for Small RNA (TaKaRa, Dalian, China). Approximately 50 mg of leaf tissue was ground into a powder under liquid nitrogen and incubated with 1 ml of RNAiso reagent. The tubes were shaken vigorously by hand for 30 seconds and incubated for 5 minutes prior to centrifugation at 12,000 g for 15 minutes at 4°C. The aqueous (upper) phase of the sample was transferred to a new RNase-free tube by carefully angling the tube at 45°. The aqueous phase was extracted with chloroform by vortexing for 2 minutes and centrifuging the mixture at 12,000 g for 5 minutes at 4°C. Small RNAs were precipitated with an equal volume of isopropanol at -20°C for at least 1 hour, followed by centrifugation at 12,000 g for 15 minutes at 4°C. The RNA pellets were washed with 200 μl of 75% ethanol and resuspended in RNase-free water. Finally, total RNA and small RNAs were quantified using a NanoDrop 2000c Spectrophotometer (Thermo Scientific, USA) and visually assessed on a 15% denaturing PAGE gel containing 7 M urea or on a 1.2% agarose gel.

### RNA transfer and UV cross-linking

The 15% denatured polyacrylamide gels containing 7 M urea were 15 cm wide, 20 cm long, and 1.5 mm thick. After polymerization at room temperature for 30 minutes, the gels were pre-run with 1× TBE at 40 mA (600 V) for 30 minutes. The RNAs or oligonucleotide probes were mixed with 2× loading buffer (5 mM EDTA, 0.1% bromophenol blue, 0.1% xylene cyanol, and 95% formamide); the RNA samples were heated at 70°C for 5 minutes. The PAGE gels were run at 40 mA (600 V) for approximately 2 hours until the bromophenol blue reached approximately 1 cm above the bottom of the gel. The gels were then stained with 0.5 μg/ml EtBr in DEPC-treated TBE buffer for 10 minutes. The RNAs were then transferred onto Hybond-N + positively charged nylon membranes (GE healthcare, USA) using a Bio-Rad transblot apparatus at 200 mA (9 - 10 V) for 3 hours (Bio-Rad, USA). Next, the membranes were further crosslinked at 1200 μjoules for 20 minutes and dried at 50°C for 30 minutes to improve sensitivity. Membranes prepared in this way can be stored at 4°C for several months.

### Hybridization

Before hybridization, the membranes were pre-hybridized for at least 30 minutes at 40°C in pre-hybridization buffer (7% SDS, 200 mM Na_2_HPO_4_ (pH 7.0), 5 μg/ml salmon sperm DNA (SSDNA)). A 3-hour incubation period is recommended for decreasing background noise. Next, the pre-hybridization buffer was removed, and hybridization buffer containing 50 pmol/ml labeled probes was added. The biotin-labeled probe did not require denaturation. The membranes were hybridized for 12 to 16 hours at 40°C with gentle shaking and subsequently rinsed with washing buffer (1× SSC, 0.1% SDS) 3 times for approximately 15 minutes total at room temperature.

### ECL lighting

The biotin-labeled probes were detected using a Chemiluminescent Nucleic Acid Detection Module Kit (Thermo Scientific, USA). After rinsing, the membranes were blocked again with blocking buffer for 15 minutes with gentle shaking at room temperature followed by incubation for an additional 15 minutes with hybridization buffer containing stabilized streptavidin-HRP conjugate. After washing 3 times, the membranes were equilibrated in substrate equilibration buffer for 5 minutes. Finally, the membranes were placed in a clean container, covered completely with working solution, and incubated for 5 minutes in the dark. A working solution was prepared with luminol/enhancer solution and stable peroxide solution. The membranes were placed in a cassette with X-ray films and exposed for different periods of time (depending on the desired signal intensity).

## Authors′ contributions

QH performed the experiments and analyzed the data, ZM performed the experiments and analyzed the data, SL performed the experiments and analyzed the data, JH conceived the study, performed the experiments, analyzed the data and wrote the manuscript, YZ conceived the study. All authors read and approved the final manuscript.

## Additional file

## Electronic supplementary material

Additional file 1: Table S1.: Probes used in this study. (DOC 33 KB)

Below are the links to the authors’ original submitted files for images.

Authors’ original file for figure 1

Authors’ original file for figure 2

Authors’ original file for figure 3

Authors’ original file for figure 4
