# Supplementary material for: A non-radioactive method for small RNA detection by northern blotting
Source: Rice (N Y). 2014 Oct 1;7:26. doi: 10.1186/s12284-014-0026-1 (PMC4884002; doi:10.1186/s12284-014-0026-1)
Supplement: Supplementary file 1 — Additional file 1: Table S1.: Probes used in this study. (DOC 33 KB) [file 12284_2014_26_MOESM1_ESM.doc]

Supplemental Table 1. Probes used in this study

| ID | Sequences | Purposes |
| --- | --- | --- |
| miR5078 | AAATGCCACGGTCGAACGAGG | Rice |
| miR156 | GTGCTCACTCTCTTCTGTCA | Rice and Arabidopsis |
| miR171b-Rice | GATATTGGCACGGCTCAATCA | Rice |
| miR171a-Ara | GATATTGGCACGGCTCAATCA | Arabidopsis |
| miR390b | GGCGCTATCCCTCCTGAGCTT | Rice and Arabidopsis |
| miR168a-Rice | GTCCCGATCTGCACCAAGCGA | Rice |
| miR168a-Ara | TTCCCGACCTGCACCAAGCGA | Arabidopsis |
| ta-siR255 | TACGCTATGTTGGACTTAGAA | Rice and Arabidopsis |
| ta-siR752 | AACGCTATGTTGGACTTAGGA | Rice and Arabidopsis |
| ta-siR850 | GTCGATATGTTGAACTTAGAA | Rice and Arabidopsis |
| ta-siR2141 | AAGGCCTTACAAGGTCAAGAA | Rice and Arabidopsis |
