# Supplementary figures and images for: A non-radioactive method for small RNA detection by northern blotting
Source: Rice (N Y). 2014 Oct 1;7:26. doi: 10.1186/s12284-014-0026-1 (PMC4884002; doi:10.1186/s12284-014-0026-1)

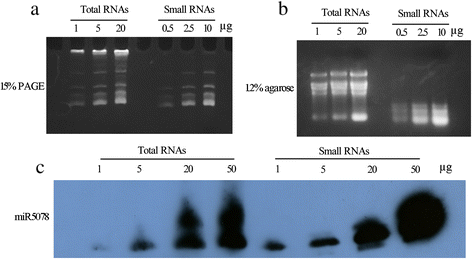

Supplement: Supplementary file 2 — Authors’ original file for figure 1 [file 12284_2014_26_MOESM2_ESM.gif]

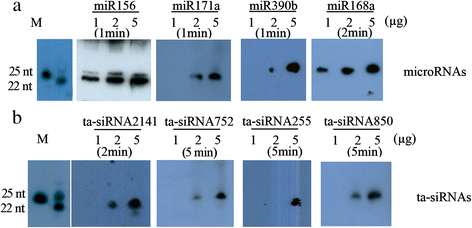

Supplement: Supplementary file 3 — Authors’ original file for figure 2 [file 12284_2014_26_MOESM3_ESM.gif]

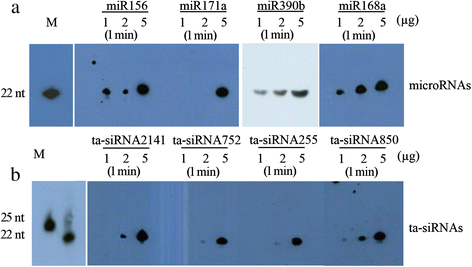

Supplement: Supplementary file 4 — Authors’ original file for figure 3 [file 12284_2014_26_MOESM4_ESM.gif]

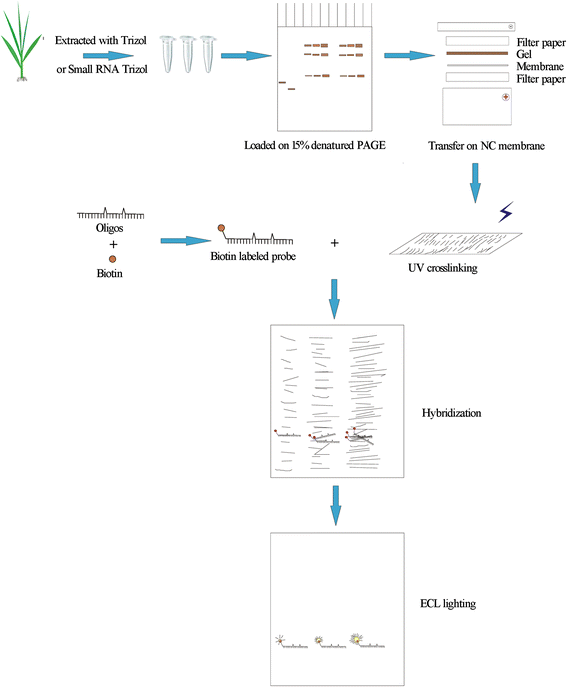

Supplement: Supplementary file 5 — Authors’ original file for figure 4 [file 12284_2014_26_MOESM5_ESM.gif]
